# Stress-induced alterations of mesocortical and mesolimbic dopaminergic pathways

**DOI:** 10.1038/s41598-021-90521-y

**Published:** 2021-05-26

**Authors:** F. Quessy, T. Bittar, L. J. Blanchette, M. Lévesque, B. Labonté

**Affiliations:** 1 CERVO Brain Research Centre, Quebec, QC Canada; 2 Department of Psychiatry and Neuroscience, Faculty of Medicine, Université Laval, Quebec, QC Canada

**Keywords:** Neural circuits, Stress and resilience

## Abstract

Our ability to develop the cognitive strategies required to deal with daily-life stress is regulated by region-specific neuronal networks. Experimental evidence suggests that prolonged stress in mice induces depressive-like behaviors via morphological, functional and molecular changes affecting the mesolimbic and mesocortical dopaminergic pathways. Yet, the molecular interactions underlying these changes are still poorly understood, and whether they affect males and females similarly is unknown. Here, we used chronic social defeat stress (CSDS) to induce depressive-like behaviors in male and female mice. Density of the mesolimbic and mesocortical projections was assessed via immuno-histochemistry combined with Sholl analysis along with the staining of activity-dependent markers pERK and c-fos in the ventral tegmental area (VTA), nucleus accumbens (NAc) and medial prefrontal cortex (mPFC). Our results show that social stress decreases the density of TH^+^ dopaminergic axonal projections in the deep layers of the mPFC in susceptible but not resilient male and female mice. Consistently, our analyses suggest that pERK expression is decreased in the mPFC but increased in the NAc following CSDS in males and females, with no change in c-fos expression in both sexes. Overall, our findings indicate that social defeat stress impacts the mesolimbic and mesocortical pathways by altering the molecular interactions regulating somatic and axonal plasticity in males and females.

## Introduction

Major depressive disorder (MDD) is a complex and highly heterogeneous mental disorder affecting yearly more than 267 million people worldwide^1^. This heterogeneity, combined with complex environmental, genetic and molecular etiologies, interferes with our capacity to treat MDD efficiently, and consequently imposes major economic and medical burden on modern societies^2^. Part of the complexity of MDD emerges from its sexually dimorphic nature. In Canada, the incidence of MDD is 1.7-fold greater in women compared to men^3^. Clinical studies report women to exhibit: higher scores of depression, younger age of onset, higher number of depressive episodes and relapse rate^4–7^. Furthermore, differences in treatment response to antidepressants have been reported between men and women with MDD^8,9^. Together, this suggests that the functional and molecular mechanisms underlying the expression of the disease may differ significantly in men and women, although the nature of these differences and their contribution to the expression of MDD in both sexes remain poorly understood.

Historically, the implication of monoamines, including dopamine (DA), has dominated the field of MDD research showing the key roles DA circuits on mood and motivation^10^. DA neurons in the brain are mainly found in the ventral tegmental area (VTA) and the substantia nigra pars compacta (SNpc). There is a large body of evidence that shows molecular, functional, and transcriptional alterations of the DA system induced by chronic stress. For instance, human studies associated single nucleotide polymorphisms (SNPs) on dopaminergic genes with elevated risk to develop MDD^11,12^. Lower expression of the dopamine transporter (DAT) has also been reported in the brain of MDD patients^13,14^ and mice susceptible to social stress^15–17^. Furthermore, impaired binding of DA to dopamine receptors 1 and 2 (DRD) has been reported in the striatum of depressed suicide completers^18^, although DA levels are not modified in suicide completers^19–21^. Furthermore, the contribution of DRD2 in mediating susceptibility to social stress has also been widely investigated in rodents^22–24^.

Importantly, DA neurons from the VTA project to several parts of the brain to modulate a wide spectrum of emotionally-relevant behavioral processes such as reward, salience, fear, aversion and memory^25–27^: many of which have been shown to be impaired in MDD patients and mouse models of chronic stress^28–30^. Amongst these different DA circuits, the mesolimbic and mesocortical pathways have been consistently associated with the expression of stress responses in mice^31–33^. For instance, previous functional studies showed that optogenetic activation of the mesolimbic pathway induces stress susceptibility, while its inhibition promotes resilience to social stress^34^. In contrast, inhibition of the mesocortical pathway was shown to induce susceptibility to social stress, with no behavioral effect associated with the inhibition of this pathway in male mice^32,34^. Moreover, a study revealed opposite dopamine metabolism responses between mesocortical and mesolimbic pathways in mice undergoing the forced swim test^33^. While these results support the distinct contribution of both pathways in mediating the impact of social stress, their contribution in males and females remains unclear.

In this study, we combined morphological and molecular approaches to investigate the effects of chronic social stress on the mesolimbic and mesocortical DA circuits and compared these effects in males and females. Overall, through these approaches, we provide insights into the distinct contributions of both pathways in mediating susceptibility or resilience to social stress in males and females.

## Results

**Figure 1:**
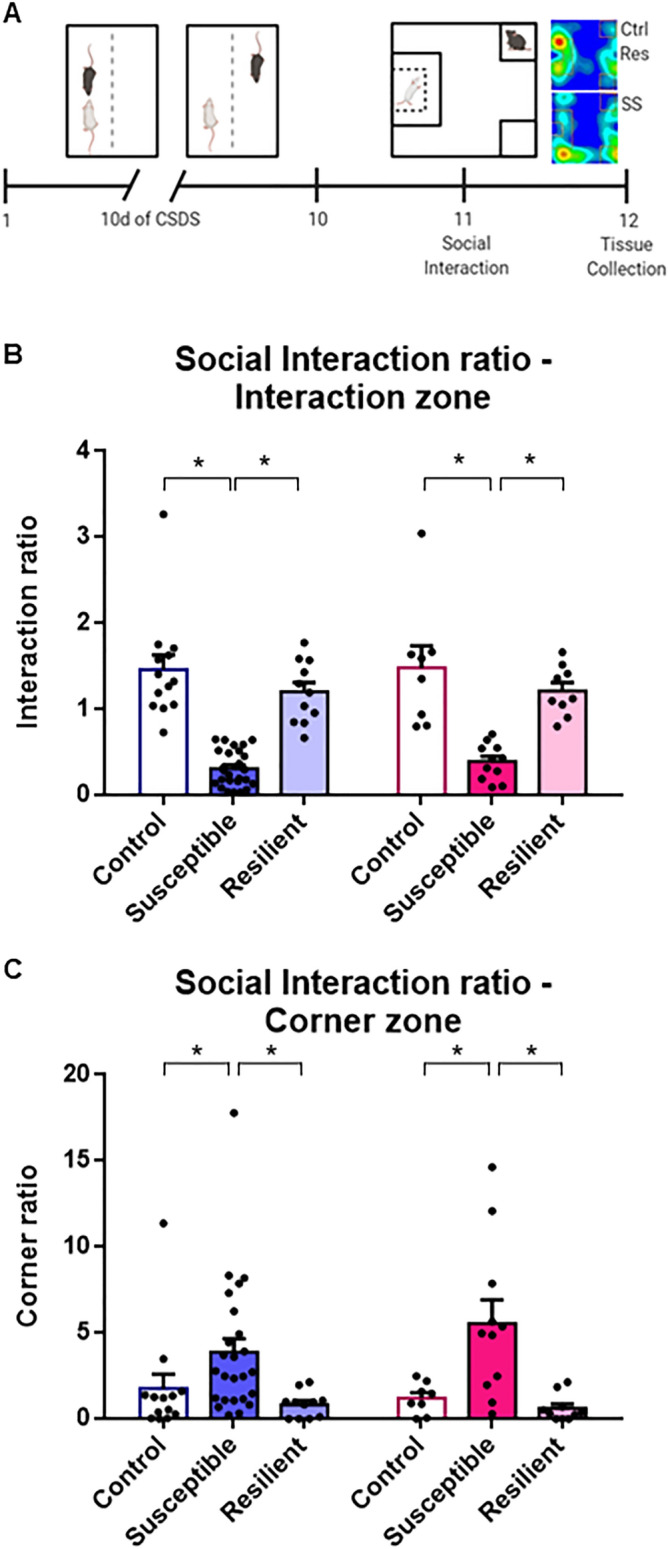
Chronic social defeat stress induces social avoidance in males and females. (**A**) Schematic diagram showing the experimental procedure for CSDS. (**B**) Repeated CSDS induces social avoidance in susceptible male and female mice but resilient mice keep interacting with CD1 aggressors [two-way ANOVA, Phenotype: F_(2,72)_ = 53.25, *p* < 0.05]. (**C**) Male and female mice susceptible to CSDS spend more time in the corners of the open field compared to resilient and control mice [two-way ANOVA, Phenotype: F_(2,72)_ = 12.41, *p* < 0.05]. n = total/male/female, control n = 21/13/8, susceptible n = 37/26/11, resilient n = 20/11/9. Bar graphs show mean ± SEM. Each dot represents a single mouse. *p ≤ 0.05.

We compared the impact of chronic social defeat stress (CSDS) on the morphological and molecular properties of the mesolimbic and mesocortical dopaminergic pathways in male and female mice (Fig. 1A). Ten days of CSDS induced social avoidance in 26 males (70%) and 11 females (55%) while the remaining 11 males (30%) and 9 females (45%) continued to interact with the CD1 target (Phenotype main effect: F_(2,72)_ = 53.25, *p* < 0.05; Fig. 1B). As expected, susceptible male and female mice spent more time in the corners of the open field, avoiding CD1 targets compared to resilient males (Phenotype main effect: F_(2,72)_ = 12.41, *p* < 0.05, Fig. 1C).

### The impact of chronic social stress on the morphological properties of DA mesolimbic and mesocortical circuits

**Figure 2:**
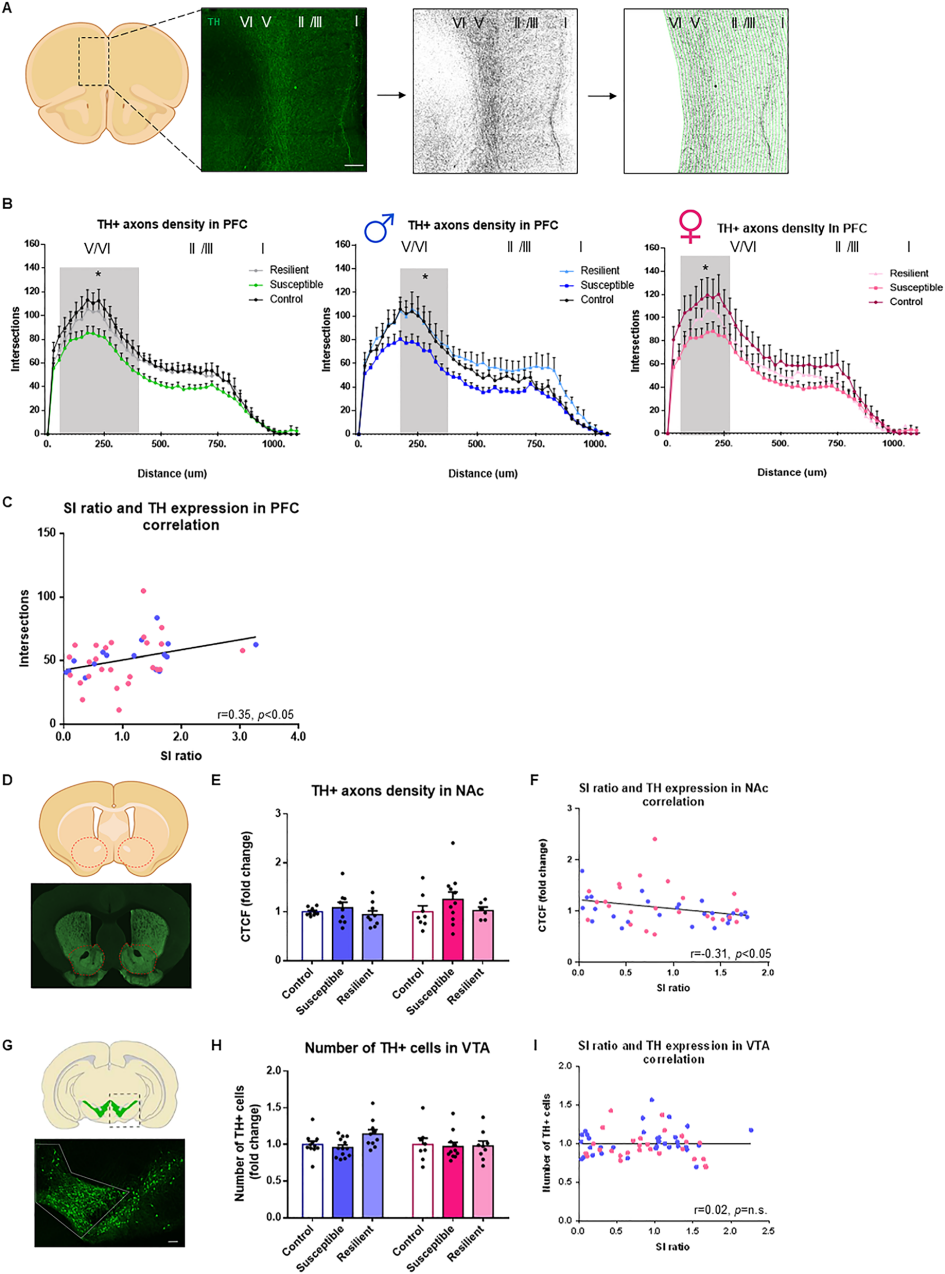
CSDS induces pathway-specific morphological changes in mesolimbic and mesocortical circuits from males and females. (**A**) Representative example of a prefrontal cortex section stained with TH antibody with its depiction for morphological analysis. Scale bar, 150 μm. (**B**) TH+ neurite density repartition in the different layers of the mPFC. Distance 0 corresponds to the corpus collosum (Layer VI) up to the medial line of the brain (Layer I) [Two-way ANOVA, Phenotype: F_(2,1616)_ = 62.61, *p* < 0.05; n = total/male/female, control n = 13/6/7, susceptible n = 17/5/12, resilient n = 11/5/6]. (**C**) Linear correlation between SI ratios and DA arborization in the mPFC [linear regression, r = 0.35; *p* < 0.05; n = 41]. (**D**) Representative example of TH immunostaining in the NAc. (**E**) Fluorescence density of TH+ axons in the NAc [Two-way ANOVA, Phenotype factor: F_(2,46)_ = 1.69, *p* = n.s; n = total/male/female, control n = 17/9/8, susceptible n = 20/9/11, resilient n = 15/9/6]. (**F**) Linear correlation between SI ratios and TH+ density in the NAc [linear regression, r = − 0.30, *p* < 0.05; n = 52]. (**G**) Representative example of TH immunostaining in the VTA. Scale bar, 100 μm. (**H**) Stereological counts of TH+ neurons in the VTA [Two-way ANOVA, Phenotype factor: F_(2,56)_ = 1.51, *p* = n.s; n = total/male/female, control n = 19/11/8, susceptible n = 24/13/11, resilient n = 19/10/9]. (**I**) Linear correlation between SI ratios and the number of TH+ neurons in the VTA [linear regression, r = 0.01, *p* = n.s; n = 61]. Bar graphs show mean ± SEM. Data are represented as fold change compared to controls. Each dot represents a single mouse. Values derived from at least three sections per brain. *p ≤ 0.05.

Our first objective was to test whether chronic social stress induces a morphological reorganization of DA circuits in males and females. We first combined IHC staining with 2D Sholl analysis^35^ to quantify variations in the axonal arborization of VTA DA neurons projecting to the different layers of the medial prefrontal cortex (mPFC) in males and females after CSDS. Consistent with previous studies^36^, our analysis of the mesocortical pathway revealed sparse DA inputs located mostly in the deep layers V and VI of the mPFC, with axonal ramifications in the more superficial layers II/III and I (Fig. 2A). Interestingly, further analyses (Phenotype main effect: F_(2,1616)_ = 62.61, *p* < 0.05) revealed a significantly smaller DA axonal density in the mPFC of both male (p < 0.05) and female (p < 0.05) susceptible mice, but not resilient, compared to controls (Fig. 2B). While consistently observed across all cortical layers, these effects are more prominent in layers V/VI of the mPFC (see Supplemental Table 1 for complete statistical details). Additionally, our analysis revealed a significant positive correlation between social interaction ratios and TH+ axonal arborization in the mPFC (r = 0.35; *p* < 0.05, Fig. 2C) suggesting that smaller arborization of DA inputs in the mPFC may promote susceptibility to CSDS in male and female mice.

We next measured DA innervation in the nucleus accumbens (NAc). Because DA inputs to the NAc are too dense to perform Sholl analysis, we quantified DA inputs by measuring corrected total cell fluorescence intensity (CTCF^37–39^; Fig. 2D). Our analysis indicated no effect of CSDS on DA axonal density in the entire NAc of males and females (Fig. 2E). However, our analysis revealed a significant negative correlation between social interaction ratios and levels of TH fluorescence (r = − 0.35, *p* < 0.05; Fig. 2F), suggesting that smaller DA axonal arborization in the NAc may promote resilience to social stress. We then analyzed TH fluorescence more specifically in the core and shell sub-regions of the NAc, since both sub-regions have been involved in the control of distinct behavioral features relevant to emotional responses to chronic stress^40–42^. Consistent with our analysis of the entire NAc structure, our analysis showed no significant difference in both shell and core regions between susceptible, resilient, and control mice in either males or females (SI Figure 1A,C). However, we found inverse correlations between social interaction ratios and TH density in both shell (r = − 0.30; *p* < 0.05, SI Figure 1B), and core (r = − 0.25; *p* < 0.1, SI Figure 1D) regions from males and females. This suggests that increased DA axonal arborization in the NAc shell or core may increase susceptibility to chronic social stress in male and female mice.

We next counted TH+ neurons in the VTA (Fig. 2G) to verify whether the changes in DA axonal innervation described above could be due to variations in the number of DA neurons in the VTA. As expected, our analysis indicated that the number of TH+ neurons in the VTA remains unchanged in susceptible and resilient male and female mice, compared to controls (Fig. 2H) with no correlation between social interaction ratios and the number of TH+ neurons in the VTA (Fig. 2I). Together, our results suggest that CSDS reduces the arborization of DA inputs from the VTA to the mPFC of males and females with no effect in the NAc.

### The impact of chronic social stress on ERK and c-fos intracellular signaling pathways in DA mesolimbic and mesocortical circuits

Our next objective was to test whether the morphological remodeling of the mesolimbic and mesocortical DA pathways induced by CSDS associates with functional changes in the activity of target brain regions by quantifying the expression of the intracellular activity markers phospho-ERK (pERK) and c-fos in the VTA, NAc, and mPFC of susceptible and resilient male and female mice.

**Figure 3:**
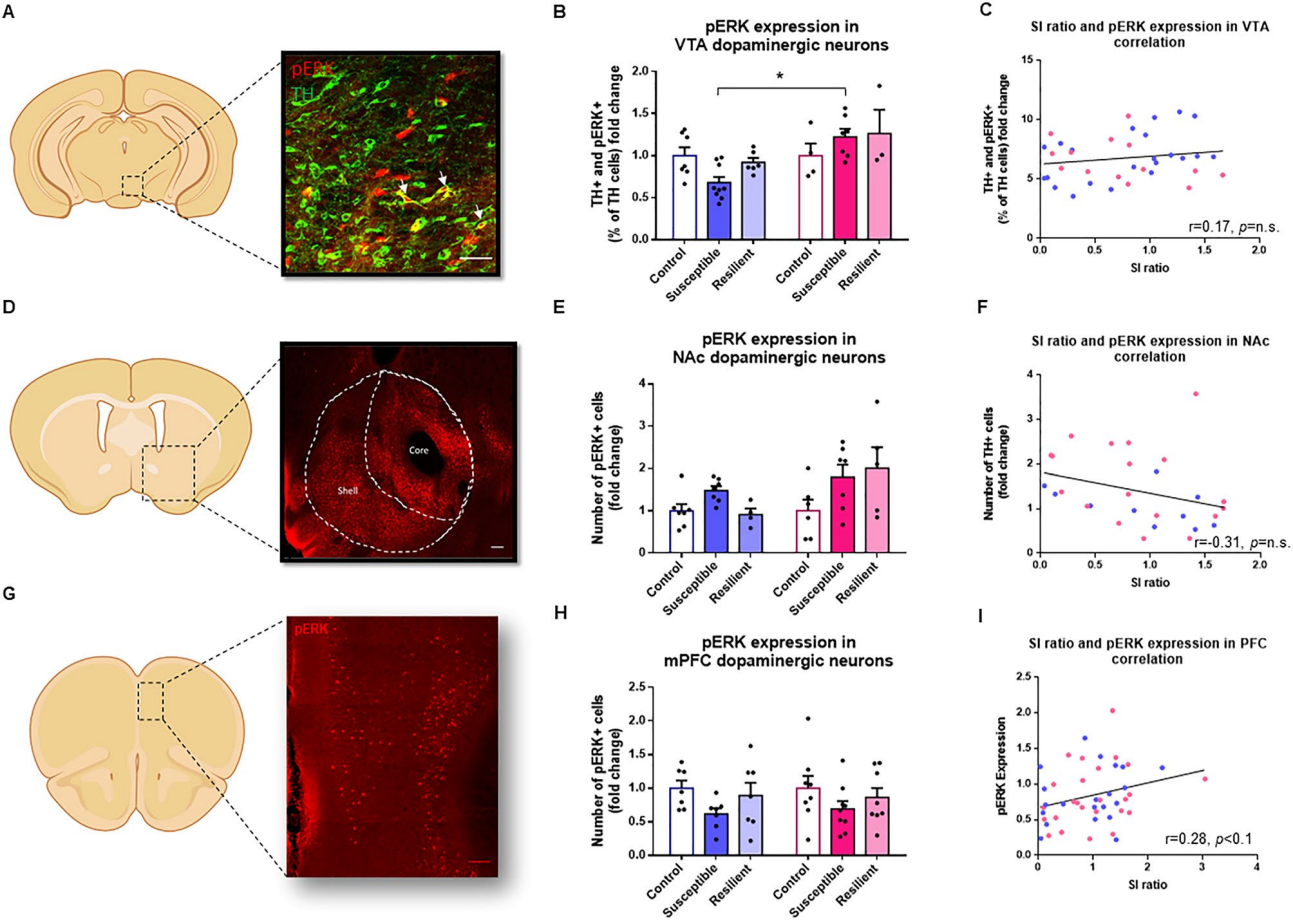
CSDS interferes with ERK signaling in target regions of the mesolimbic and mesocortical DA circuits. (**A**) Representative example of TH (green) and pERK (red) immunostaining in the VTA. Scale bar, 150 μm. (**B**) Stereological count of TH+/pERK+ neurons in the VTA [Two-way ANOVA, Sex × Phenotype Interaction main effect: F_(2,30)_ = 3.56, *p* < 0.05; n = total/male/ female, control n = 11/7/4, susceptible n = 16/9/7, resilient n = 9/6/3]. (**C**) Linear correlation between SI ratios and the number of TH+/pERK+ neurons in the VTA [linear regression, r = 0.17, *p* = n.s; n = 36]. (**D**) Representative example of pERK (red) immunostaining in the NAc delineating the shell and core subregions. Scale bar, 150 μm. (**E**) Stereological count of pERK+ neurons in the NAc [Two-way ANOVA, Phenotype main factor: F_(2,30)_ = 3.51, *p* < 0.05; n = total/male/female, control n = 13/7/6, susceptible n = 14/7/7, resilient n = 9/4/5]. (**F**) Linear correlation between SI ratios and the number of pERK+ neurons in the NAc [linear regression, r = − 0.31, *p* = n.s; n = 28]. (**G**) Representative example of pERK (red) immunostaining in the mPFC. Scale bar, 150 μm. (**H**) Stereological counts of pERK+ neurons in the mPFC [Two-way ANOVA, Phenotype factor: F_(2,40)_ = 2.94, *p* < 0.1; n = total/male/female, control n = 15/7/8, susceptible n = 16/7/9, resilient n = 15/7/8]. (**I**) Linear correlation between SI ratios and the number of pERK+ neurons in the mPFC [linear regression, r = 0.28, *p* < 0.1; n = 46]. Bar graphs show mean ± SEM. Data are represented as fold change from same group control values. Each dot represents one single mouse. Values derived from at least three sections per brain. *p ≤ 0.05.

**Figure 4:**
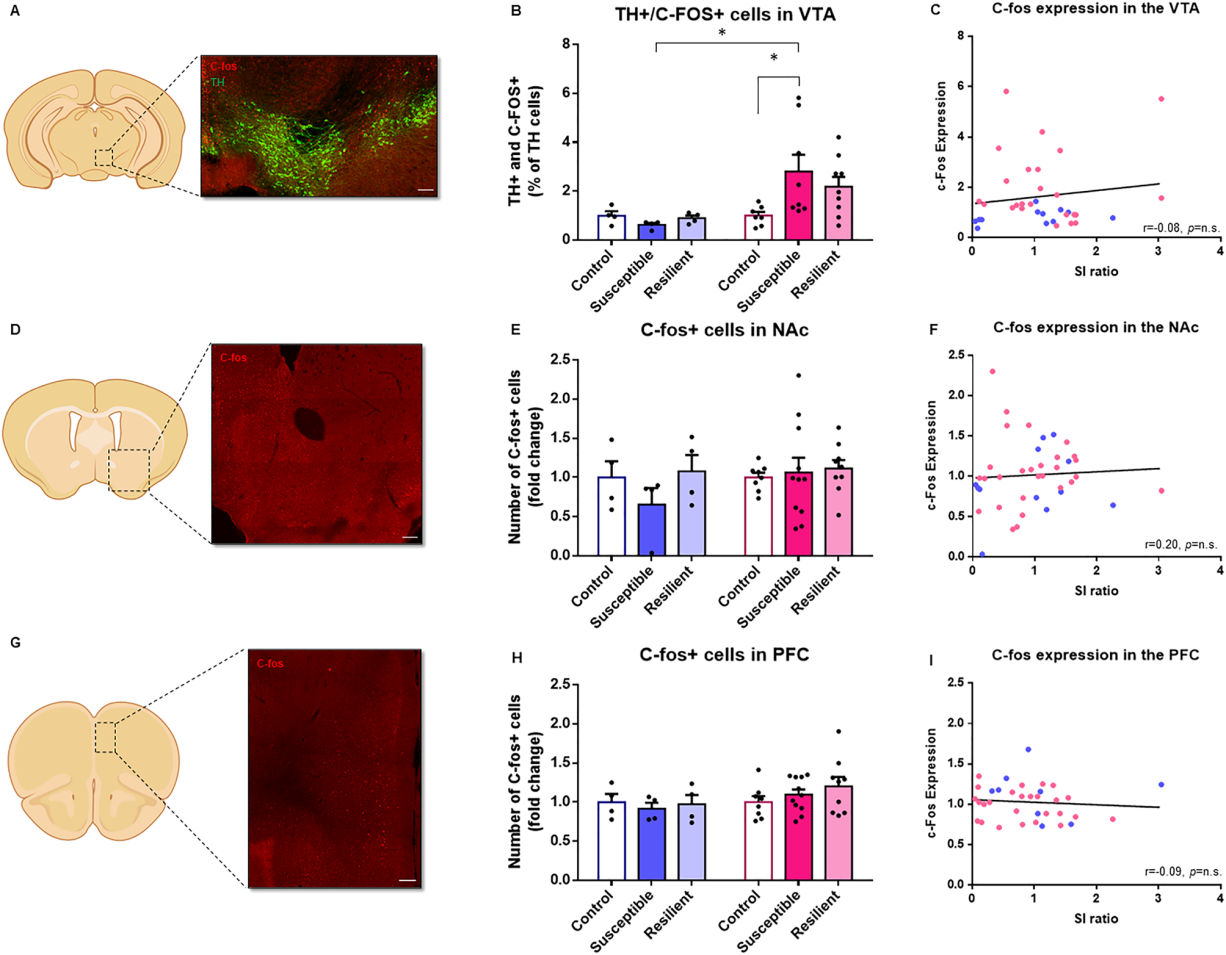
CSDS interferes with c-fos signaling in target regions of the mesolimbic and mesocortical DA circuits. (**A**) Representative example of TH+ (green) and c-fos (red) immunostaining in the VTA. Scale bar, 150 μm. (**B**) Stereological counts of TH+ and c-fos+ neurons in VTA [Two-way ANOVA, Sex × Phenotype Interaction main effect: F_(2,30)_ = 2.40, *p* = 0.1; n = total/male/female, control n = 11/4/7, susceptible n = 12/4/8, resilient n = 13/4/9]. (**C**) Linear correlation between SI ratios and the number of TH/c-fos+ neurons in the VTA [linear regression, r = 0.14, *p* = n.s; n = 36]. (**D**) Representative example of c-fos (red) immunostaining in the NAc. Scale bar, 150 μm. (**E**) Stereological counts of c-fos+ neurons in the NAc [Two-way ANOVA, Phenotype main factor: F_(2,34)_ = 0.85, *p* = n.s; n = total/male/female, control n = 12/4/8, susceptible n = 15/4/11, resilient n = 13/4/9]. (**F**) Linear correlation between SI ratios and the number of c-fos+ neurons in the NAc [linear regression, r = 0.06, *p* = n.s; n = 40]. (**G**) Representative example of pERK (red) immunostaining in the mPFC. Scale bar, 150 μm. (**H**) Stereological counts of c-fos+ neurons in the mPFC [Two-way ANOVA, Phenotype main factor: F_(2,34)_ = 0.42, *p* = n.s; n = total/male/female, control n = 12/4/8, susceptible n = 15/4/11, resilient n = 13/4/9]. (**I**) Linear correlation between SI ratios and the number of c-fos+ neurons in the mPFC [linear regression, r = − 0.09, *p* = n.s; n = 35]. Bar graphs show mean ± SEM. Data are represented as fold change from same group control values. Each dot represents one single mouse. Values derived from at least three sections per brain. *p ≤ 0.05.

We first quantified the number of pERK and c-fos positive neurons within TH+ DA neurons in the VTA (Fig. 3A). Interestingly, our analysis revealed an opposite regulation of pERK in stressed male and female TH+ neurons (Sex × Phenotype Interaction main effect: F_(2,30)_ = 3.56, *p* < 0.05), with post hoc analysis showing significantly lower pERK expression levels in susceptible males, compared to susceptible females (p < 0.05, Fig. 3B). However, we found no significant correlation between social interaction ratios and pERK expression in TH+ DA neurons (Fig. 3C). In contrast, our analysis of c-fos expression levels in TH+ DA neurons revealed a sex-specific effect (Sex × Phenotype Interaction main effect: F_(2,30)_ = 2.40, *p* = 0.1) with a significant increase in females susceptible to CSDS, compared to female controls (*p* < 0.05) and susceptible males (p < 0.05, Fig. 4B), but no correlation between c-fos expression levels and social interaction ratios in males and females (Fig. 4C).

In the NAc (Fig. 3D), our analysis showed that social stress increases pERK expression levels in susceptible mice compared to controls (Phenotype main effect: F_(2,30)_ = 3.51, *p* < 0.05) in both males and females (Fig. 3E), although these levels were not correlated with social interaction ratios in both sexes (Fig. 3F). Interestingly, investigating these effects in the shell and core regions of the NAc revealed distinct effects in both structures. For instance, our analysis in the shell revealed a sex-specific effect (Sex × Phenotype Interaction main effect: F_(2,30)_ = 3.22, *p* = 0.05) with susceptible (*p* < 0.05) and resilient (*p* < 0.05) female mice exhibiting significantly higher levels of pERK expression than controls (SI Figure 3A). Our analysis in the core revealed only a main effect of CSDS on pERK levels affecting males and females similarly (Phenotype main effect: F_(2,30)_ = 3.67, *p* < 0.05; SI Figure 3C). Further analyses identified a trend toward a negative correlation between pERK expression levels and social interaction ratios in the shell (r = − 0.35, *p* < 0.1; SI Figure 3B), but not the core (SI Figure 3D) region of the NAc in males and females. In contrast, we found no change in the expression of c-fos in either susceptible or resilient male and female mice, when compared to controls (Fig. 4E) with no correlation between c-fos levels and social interaction ratios in males and females (Fig. 4F).

Finally, the analysis of the mPFC (Fig. 3G) revealed a trend toward a significant effect of CSDS on pERK expression (Phenotype main effect: F_(2,40)_ = 2.94, *p* < 0.1; Fig. 3H) affecting males and females similarly. Further analyses identified a trend toward a significant positive correlation between pERK expression and social interaction ratios, suggesting that low levels of pERK expression in the mPFC may promote susceptibility to social stress in males and females (r = 0.28, *p* < 0.1, Fig. 3I). In contrast, our analysis revealed no change in the expression of c-fos in either susceptible or resilient male and female mice, compared to controls (Fig. 4H) with no correlation between c-fos expression levels and social interactions ratios in both sexes (Fig. 4I).

Further analyses showed that these effects are found in the infralimbic mPFC more predominantly with a trend toward a significant effect of CSDS (Phenotype main effect: F_(2,40)_ = 3.10, *p* < 0.1; SI Figure 4A) on pERK levels in males and females, with no effect in the prelimbic region (SI Figure 4B). In contrast, we found no effect of CSDS on c-fos expression in both mPFC subregions in males and females (SI Figure 6A,B). Finally, investigating these effects by layer [superficial (II–III) or deep (V–VI)] revealed a trend toward a significant effect of CSDS on pERK expression in the superficial layers (II–III) of the mPFC from males and females (Phenotype main effect: F(2,27) = 3.26, *p* < 0.1; SI Figure 4C) with no effect in deeper layers (V–VI; SI Figure 4D), and no effect in either superficial or deep layers for c-fos expression in males and females (SI Figure 6C,D). Overall, our results suggest that pERK expression is increased in the NAc and decreased in the mPFC, following CSDS in males and females. Globally, this suggests that variations in pERK, but not c-fos expression in the target brain regions of the mesolimbic and mesocortical dopaminergic pathways, may promote susceptibility to social stress in males and females.

## Discussion

Dysregulation of dopaminergic transmission has been widely involved in the pathophysiology of depression^43^. Depressive disorder symptoms such as social avoidance, anhedonia, loss of appetite, helplessness, and amotivation have been consistently associated with dysfunctions of dopamine signaling and functions^34,44–46^. Now, it is increasingly accepted that males and females respond differently to chronic stress^47,48^ suggesting that the DA system could exhibit sex-specific morphological and molecular adaptations to chronic stress in a sex-specific fashion. Here, we report findings showing morphological and molecular alterations in mesocortical and mesolimbic dopaminergic pathways of both stressed male and female mice and provide new insights into the implication of dopaminergic circuits in mediating stress susceptibility in a sex-specific fashion.

Neuroanatomical and morphological changes in several brain regions have been consistently associated with MDD. For example, post-mortem studies on MDD patients showed a decrease of neuronal soma size in layers V and VI of the anterior cingulate cortex when compared to healthy subjects^49,50^. Smaller volume of dorsal striatal gray matter was also shown to correlate with suicidal ideation in adolescents^51^. Similarly, mice susceptible to social stress exhibit lower spine density in the mPFC and increased spine density in the NAc and the VTA^52^ associated with higher and lower volume in the VTA and NAc, respectively^53^. Here, we provide results showing that CSDS does not change the number of DA cells in the VTA, but rather induces an important remodelling of the DA mesocortical and mesolimbic circuits in males and females. In the NAc, our findings suggest that chronic social stress does not change DA axon density in the shell nor in the core, although DA axonal density in both sub-regions are inversely correlated with the proportion of male and female mice to interact with social targets. DA projections from the VTA target mainly the shell part of the NAc composed of medium spiny neurons (MSN) expressing either DRD1, DRD2 or both receptors^54,55^. Importantly, lower frequency of excitatory inputs onto DRD1, and higher frequency onto DRD2 expressing MSNs, has been previously reported in the NAc of susceptible mice to CSDS. Optogenetic stimulations of DRD1 MSNs was associated with the expression of resilience, while the optogenetic inhibition of the same MSNs was shown to induce depressive-like behaviors following CSDS^56^. Accordingly, higher DA axonal density in the NAc shell and core subregions could cause an imbalance in dopamine regulation in this region and promote some of the behavioral deficits induced by CSDS in males and females. More work will be required to determine whether these changes impact DA transmission at DRD1 or DRD2 MSNs more specifically.

In contrast, our findings in the mPFC indicate that CSDS induces a drastic reduction of DA inputs from the VTA in susceptible male and female mice. DA neurons from the VTA project mainly in layers V and VI of the mPFC^57^. Interestingly, optogenetic inhibition of the mesocortical DA pathway was previously shown to promote susceptibility to CSDS in males^34^. Functionally speaking, the lower density of DA axons observed in our study is likely to decrease DA signaling in this region and promote social withdrawal in males and females after CSDS. Importantly, while providing interesting insights into the morphological impact of CSDS on DA circuits, the approach that uses immunohistochemistry to label TH carries limitations that could interfere with the interpretation of our results. For instance, it has been estimated that 10% of TH+ axons in the mPFC originates from axons from the locus coeruleus (LC)^58^. However, mPFC inputs from the LC are mainly located in layers II/III^36^ and as such, may not interfere with our main findings in layers V/VI. Nevertheless, the results of our study suggest that CSDS induces morphological alterations in DA axons, and could lead to distinct functional impairments in dopaminergic signaling in the NAc and mPFC.

Importantly, our findings show that morphological changes in DA circuits induced by chronic social stress associate with molecular changes affecting intracellular signaling in DA neurons and their targeted brain regions. Transcriptional profiling studies previously showed that chronic stress that includes CSDS, induces a global reorganization of transcriptional structures of DA neurons and its targeted brain regions^59,60^. Importantly, these alterations have also been associated with changes within several intracellular signaling pathways including MAPK^41,61,62^, AKT^63,64^, mTOR^65^ and GTPase pathways^66,67^. Of particular interest, molecular markers such as pERK and c-fos have been used as proxies of neuronal activity in several mouse models of chronic and acute stress^47,61,68^. Here, we found an increase in c-fos expression in the VTA of susceptible female mice which is consistent with previous observations in females after CSDS^47^ and supports the sex-specific involvement of this intracellular signaling pathway in the processing emotional responses to social cues^69^. Consistently, we provide evidence for an opposite regulation of pERK expression in susceptible male and female mice. This is in contrast with results from previous studies that showed elevated ERK activity in neurons of the VTA after CSDS^61,62^. Noticeably, the effects reported in our study were observed in a small proportion of DA neurons in the VTA (approx. 6%), while most of the pERK+ neurons in the VTA did not express TH, which could explain the discrepancies between our study and others. Thus, it is likely that the impact of CSDS on ERK signaling in the VTA may be relevant to the activity of different neuronal populations with distinct neuronal connectivity patterns. Previous studies have showed VTA dopamine neurons are heterogenous. Some dopaminergic neurons can release GABA and glutamate in addition to dopamine. Moreover, it has been showed that non-dopamine-releasing projection neurons in the VTA have a roles in reinforcement, motivation and learning^70^. Also, mapping studies showed that dopaminergic neurons exhibit different topological molecular signatures in the VTA^71^. Thus, the complex heterogeneity of dopamine neurons suggests that the different subtypes of neuron in VTA could play different roles in the predisposition to stress.

Functionally, the morphological changes observed in mPFC projections along with the molecular changes in pERK and c-fos expression in the VTA following CSDS are likely to impact DA signaling in a pathway-specific fashion, although indirectly. For instance, our results in the NAc suggest that CSDS increases pERK levels in the shell of susceptible female mice. Previous studies showed that stress activates mainly the shell structure of the NAc^72,73^ while drug seeking activates the core structure^74^. Consequently, the distinct pERK expression patterns observed in the core and shell suggest that both structures integrate emotional stressors differently in males and females, and suggest that distinct neuronal circuits relevant to these sub-regions in the NAc may contribute differently to the expression of stress responses in males and females. In the mPFC, our findings support a downregulation of ERK signaling in susceptible mice as shown before after CSDS^75^. Interestingly, ERK activation and signaling contributes to upregulation of dendritic spine density in the mPFC and improves learning and memory^75,76^. However, our findings are also in contrast with other studies that showed a significant increase in pERK expression in females following chronic variable stress^77^ and suggest that different types of stress may impose distinct impacts on the activity of the mPFC in males and females. Importantly, part of these discrepancies can be explained by the nature of both stress paradigms, CSDS reproduced psychosocial stress, while CVS composed physical stressors^78^. Together, this suggests that the pathways-specific morphological and molecular changes affecting the mesolimbic and mesocortical pathways in males and females interfere indirectly with DA signaling in the mPFC and NAc, although more work is required to determine the functional impact of these changes in males and females.

To conclude, results from our study provide new insights on the impact of CSDS on dopaminergic sub-circuits and the contribution of mesolimbic and mesocortical pathways on the expression of depressive-like behaviors associating these effects with morphological and molecular alterations in males and females. By doing so, we increase our understanding of the functional and molecular mechanisms from which sexual dimorphism emerge, to define the clinical characteristics of mood disorders in men and women.

## Methods

### Animals

C57bl6 male (*n* = 50) and female (*n* = 28) mice aged 7–8 weeks old and CD1 males aged 4–6 months old were obtained from Charles River Laboratories (USA). In total 50 males and 28 females were subjected to social defeat. Mice were housed under a 12 h light/dark cycle at 22–24 °C with no water and food restriction. CD1 mice were individually housed except during social defeats. All other mice were group-housed (4/cage) before and singly housed after social defeats. Laval’s University Institutional Animal Care Committee, in respect with the Canadian Council on Animal Care guidelines, approved all experimental procedures. The study was carried out in compliance with the ARRIVE guidelines (http://www.nc3rs.org.uk/page.asp?id=1357).

### Chronic social defeat stress

CSDS was performed as described before in males^79^ and females^80^. Briefly, in males, C57bl6 mice were first introduced to unknown aggressive retired CD1 breeders for a period of 5 min, during which the C57bl6 were attacked by the CD1 resident mouse. Following this initial phase, C57bl6 mice were moved to the other side of the divider for 24 h allowing a continuous sensory contact with the CD1 mouse without physical harm. The same procedure was repeated over 10 days with new unknown CD1 mice every day.

A very similar approach was used in females, with the difference that the base of the tail and the pubis of the C57bl6 female mice were soaked with 30 µl of male urine before each defeat bouts. All C57bl6 female mice were associated with different male urine so that CD1 mice never encountered the same urine twice. Urine collection was performed 1-week preceding social defeat stress with metabolic cages (Tecniplast Group, Tecniplast Canada, Canada). Male mice were housed in metabolic cages overnight. Urine was collected each morning and stored at 4 °C after being filtered. The 5-min social stress bouts were interrupted every time CD1 male mounted the C57bl6 female mice. Both male and female controls were housed 2/cage separated by a Plexiglas divider and housed in the same room.

### Social interaction

Social-avoidance behavior was assessed with the social interaction test (SI) 24 h after the end of the social defeat paradigm as described before^79,80^. One hour before the social interaction test, C57bl6 male and female mice were habituated to the testing room illuminated by red light. Briefly, SI test consisted of two phases of 150 s each. In the first phase, C57bl6 mice explored the arena (26.7 × 48.3 cm × 15.2 cm) with no target CD1 aggressor in the social interaction zone (14 × 24 cm). This initial phase was followed by a second exploratory phase but in the presence of an unknown target CD1 aggressor maintained into a mesh-wired enclosure within the social interaction zone. Time spent in the different zones of the arena was automatically recorded through ANY-Maze 4.99 using a top-view camera (ANY-Maze Video Tracking Software, Stoelting Co., USA). Based on social interaction ratios (time in interaction zone with social target/time in interaction zone without social target), defeated mice were designated as susceptible or resilient: susceptible ratio < 1.0; resilient ratio > 1.0. Corner zone (Dimensions: 9 × 9 cm) ratio was also assessed. This measure of susceptibility versus resilience has been shown to correlate with other defeat-induced behavioral abnormalities such as anhedonia (e.g. decrease in sucrose preference) and an increased sensitivity to inescapable stress^81–83^. Following the social interaction test, C57bl6 mice were housed individually for 24 h before euthanizing and tissue collection.

### Perfusions

For morphological and molecular analyses, mice were anesthetized with a lethal dose of 20% (w/v) urethane (Sigma, 51-79-6) and perfused with 40 ml of 0.1 M/L phosphate-buffered saline and 40 ml of 4% (w/v) PFA (Sigma, 30525-89-4) 24 h after social interaction. Brains were post-fixed for 24 h in PFA 4% and cryoprotected in sucrose 30% before being stored in OCT (Tissue-Tek^®^, 4583) at − 80 °C. Brains were sliced on a Leica CM1900 cryostat at 40 µm.

### Immunohistochemistry

Free-floating sections were washed with PBS and then blocked with 1% normal donkey serum and 0.2% Triton X-100 for 2 h. Brains sections were incubated overnight at 4 °C with polyclonal antibodies against tyrosine hydroxylase (TH) 1:500 (Pel-Freez Biologicals, P60101), Phospho-p44/42 MAPK 1:250 (Erk1/2) (Thr202/Tyr204) (Cell Signaling, 9101) or c-fos 1:500 (Abcam, Ab190289). The next day, after washes, brain sections were incubated in corresponding secondary antibodies (Alexa Fluor 488 Donkey anti-rabbit IgG 1:400, Life Technologies A-21206; Alexa Fluor 555 Donkey anti-sheep IgG 1:400, Life Technologies A-21099; Alexa Fluor 647 Donkey anti-rabbit IgG 1:400, Life Technologies A-31573) for 1 h at room temperature. Sections were mounted with anti-fade solution, including 40,6-diamidino-2-phenylindole (Abcam, Ab104139). A Zeiss LSM700 confocal microscope was used for TH morphological and pERK/c-fos colocalization expression for VTA analysis, and a Huron digital pathology confocal slide scanner was used to acquire the immunofluorescence images for TH expression in NAc.

All immunofluorescence images were analyzed using ImageJ Software^84^. TH, pERK and c-fos expression was assessed via counts for each region of interest (VTA, NAc, mPFC), with a minimum of 3 brain section per mouse. Brain sections were selected using the Allan Brain Atlas^85^. mPFC sections were between 1.42 and 2.245 mm of Bregma, NAc between 0.845 and 1.545 mm of Bregma and VTA between − 2.78 and − 3.38 mm of Bregma. The regions and sub-regions of interest were delimited using Allan Brain atlas reference^85^. TH+, pERK+ and c-fos+ cells were counted manually using Image J software^84^. TH density in the NAc and c-fos expression in the NAc and mPFC was analyzed using corrected total cell fluorescence method (CTCF)^37,39,86^. Expression of TH morphological arborization in prefrontal cortex was assessed using the software Neurite-J 1.1^35^.

### Statistical analysis

Statistical analyses were performed using GraphPad Prism 6.0 (GraphPadSoftware, La Jolla, CA, USA) software. Details of every statistical analysis are available in SI Tables 1–10. In total, we used 50 male and 28 female mice for analyses. Each point represents one mouse. All data are represented as means ± SEM, and significance is defined as *p ≤ 0.05.

For social interaction and corner ratios (Fig. 1), 50 male mice and 28 females were analyzed. Normality was assessed using D’Agostino and Pearson omnibus test. Group differences in social interaction and corner ratios were tested by means of two-way ANOVAs with Sex, Phenotype and Sex × Phenotype Interaction as independent variables followed by Tukey’s post hoc tests. Each point represents one mouse. All data are represented as mean ± SEM and significance is defined as *p ≤ 0.05.

For TH density analysis in the mPFC (Fig. 2A,B), 16 male mice and 26 females were analyzed. Using Grubb test on the mean of all distances with criteria of more than 1.96 standard deviations from the mean, one control female was discarded. Normality of data was assessed using D’Agostino and Pearson omnibus test. Group differences in distance and TH+ intersections were tested by means of two-way ANOVA with Sex, Phenotype and Sex × Phenotype Interaction as independent variables, followed by Tukey’s post hoc tests. All data are represented as mean ± SEM, and significance is defined as *p ≤ 0.05. Relationships between TH+ intersections and social interaction ratios were tested by means of linear regression analyses (Fig. 2C). Normality of data was assessed using Shapiro–Wilk normality test. Correlation between SI and TH density was analyze using Pearson test with two-tailed p-value. Each point represents one mouse with at least three sections of mPFC. The data were generated from 4 different groups of socially defeated mice.

For TH density analysis in the NAc (Fig. 2D,E), 27 males and 25 females were analyzed. No outlier was identified. Normality was assessed using D’Agostino and Pearson omnibus test. Group differences in TH+ axon density were tested by means of two-way ANOVA with Sex, Phenotype and Sex × Phenotype Interaction as independent variables followed by Tukey’s post hoc tests. All data are expressed in fold change over respective control groups. All data are represented as mean ± SEM and significance is defined as *p ≤ 0.05. Relationships between TH+ axon density in the NAc and social interaction ratios were tested by means of linear regression analyses (Fig. 2F). Normality of data was assessed using Shapiro–Wilk normality test. Correlation between SI and TH density was analyze using Spearman correlation test with two-tailed p-value. Each point represents one mouse with at least three sections of NAc. The mean of all sections was used to generate each point. The data were generated from 5 different groups of socially defeated mice.

For TH+ cell analysis in the VTA (Fig. 2G,H), 22 males and 28 females were analyzed. No outlier was identified. Normality was assessed using D’Agostino and Pearson omnibus test. Group differences in TH+ cell density were tested by means of two-way ANOVA with Sex, Phenotype and Sex × Phenotype Interaction as independent variables followed by Tukey’s post hoc tests. All data are expressed in fold change over their respective control groups. All data are represented as mean ± SEM and significance is defined as *p ≤ 0.05. Relationships between TH+ cell density in the VTA and social interaction ratios were tested by means of linear regression analyses (Fig. 2F). Normality of data was assessed using Shapiro–Wilk normality test. Correlation between SI and TH density was analyze using Spearman correlation test with two-tailed p-value. Each point represents one mouse with at least three sections of VTA. The mean of all sections was used to generate this point. The data were generated from four different groups of socially defeated mice.

For pERK analysis in the VTA (Fig. 3A,B), 22 males and 14 females were analyzed. No outlier was identified. Normality was assessed using D’Agostino and Pearson omnibus test. Group differences in pERK expression were tested by means of two-way ANOVA with Sex, Phenotype and Sex × Phenotype Interaction as independent variables followed by Tukey’s post hoc tests. All data are expressed in fold change over their respective control groups. All data are represented as mean ± SEM and significance is defined as *p ≤ 0.05. Relationships between pERK expression in TH+ neurons in the VTA and social interaction ratios were tested by means of linear regression analyses (Fig. 3C). Normality of data was assessed using Shapiro–Wilk normality test. Correlation between SI and pERK expression was analyze using Pearson test with two-tailed p-value. Each point represents one mouse with at least three sections of VTA. The mean of all sections was used to generate this point. The data were generated from four different groups of socially defeated mice.

For pERK expression analysis in the NAc (Fig. 3D,E), 18 males and 18 females were analyzed. No outliers were identified. Normality of data was accessed using D’Agostino and Pearson omnibus test. Group differences in pERK expression were tested by means of two-way ANOVA with Sex, Phenotype and Sex × Phenotype Interaction as independent variables followed by Tukey’s post hoc tests. All data are expressed in fold change over their respective control groups. All data are represented as means ± SEM and significance is defined as *p ≤ 0.05. Relationships between pERK expression in the NAc and social interaction ratios were tested by means of linear regression analyses (Fig. 3F). Normality of data was assessed using Shapiro–Wilk normality test. Correlation between SI and pERK expression was analyze using Pearson test with two-tailed p-value. Each point represents one mouse with at least three sections of NAc. The mean of all sections was used to generate this point. The data were generated from four different groups of socially defeated mice.

For pERK expression analysis in the mPFC (Fig. 3G,H), 21 males and 25 females were analyzed. No outlier was identified. Normality was assessed using D’Agostino and Pearson omnibus test. Group differences in pERK expression were tested by means of two-way ANOVA with Sex, Phenotype and Sex × Phenotype Interaction as independent variables followed by Tukey’s post hoc tests. All data are expressed in fold change over their respective control groups. All data are represented as means ± SEM and significance is defined as *p ≤ 0.05. Relationships between pERK expression in the mPFC and social interaction ratios were tested by means of linear regression analyses (Fig. 3I). Normality of data was assessed using Shapiro–Wilk normality test. Correlation between SI and pERK expression was analyze using Pearson test with two-tailed p-value. Each point represents one mouse with at least three sections of mPFC. The mean of all sections was used to generate this point. The data were generated from 4 different groups of social defeat.

For c-fos expression analysis in the VTA (Fig. 4A), 12 males and 24 females were analyzed. No outlier was identified. Normality was accessed using D’Agostino and Pearson omnibus test. Group differences in c-fos expression were tested by means of two-way ANOVA with Sex, Phenotype and Sex × Phenotype Interaction as independent variables followed by Tukey’s post hoc tests. All data are expressed in fold change over their respective control groups. All data are represented as means ± SEM and significance are defined as *p ≤ 0.05. Relationships between c-fos expression in the VTA and social interaction ratios were tested by means of linear regression analyses (Fig. 4B). Normality of data was assessed using Shapiro–Wilk normality test. Correlation between SI and pERK expression was analyze using Spearman test with two-tailed p-value. Each point represents one mouse with at least three sections of VTA. The mean of all sections was used to generate this point. The data were generated from 3 different groups of socially defeated mice.

For c-fos expression analysis in the NAc and mPFC (Fig. 4C–E), 12 males and 28 females were analyzed. Normality was accessed using D’Agostino and Pearson omnibus test. No outlier was identified. Group differences in c-fos expression were tested by means of two-way ANOVA with Sex, Phenotype and Sex × Phenotype Interaction as independent variables followed by Tukey’s post hoc tests. All data are expressed in fold change over their respective control groups. All data are represented as means ± SEM and significance are defined as *p ≤ 0.05. Relationships between c-fos expression in the NAc or mPFC and social interaction ratios were tested by means of linear regression analyses (Fig. 4D,F). Normality of data was assessed using Shapiro–Wilk normality test. Correlation between SI and pERK expression was analyze using Pearson (NAc) and Spearman (mPFC) tests with two-tailed p-value. Each point represents one mouse with at least three sections of NAc or mPFC. The mean of all sections was used to generate this point. The data were generated from 3 different groups of socially defeated mice.

## Supplementary Information

Supplementary Figures.

Supplementary Tables.

## Data Availability

The datasets generated during and/or analyzed during the current study are available from the corresponding author on reasonable request.
