# Supplementary material for: Stress-induced alterations of mesocortical and mesolimbic dopaminergic pathways
Source: Sci Rep. 2021 May 26;11:11000. doi: 10.1038/s41598-021-90521-y (PMC8154906; doi:10.1038/s41598-021-90521-y)
Supplement: Supplementary file 1 — Supplementary Figures. [file 41598_2021_90521_MOESM1_ESM.docx]

**Stress-induced alterations of mesocortical and mesolimbic dopaminergic pathways**

Quessy F^1,2^, Bittar T^1,2^, Blanchette LJ^1,2^, Lévesque M^1,2*^, Labonté B^1,2*^

1. CERVO Brain Research Centre, Québec, QC, Canada

2. Department of Psychiatry and Neuroscience, Faculty of Medicine, Université Laval, Québec, QC, Canada

* Equal contribution

Correspondence and requests for material should be addressed to [benoit.labonte@fmed.ulaval.ca](mailto:benoit.labonte@fmed.ulaval.ca) or [martin.levesque@fmed.ulaval.ca](mailto:martin.levesque@fmed.ulaval.ca)

**
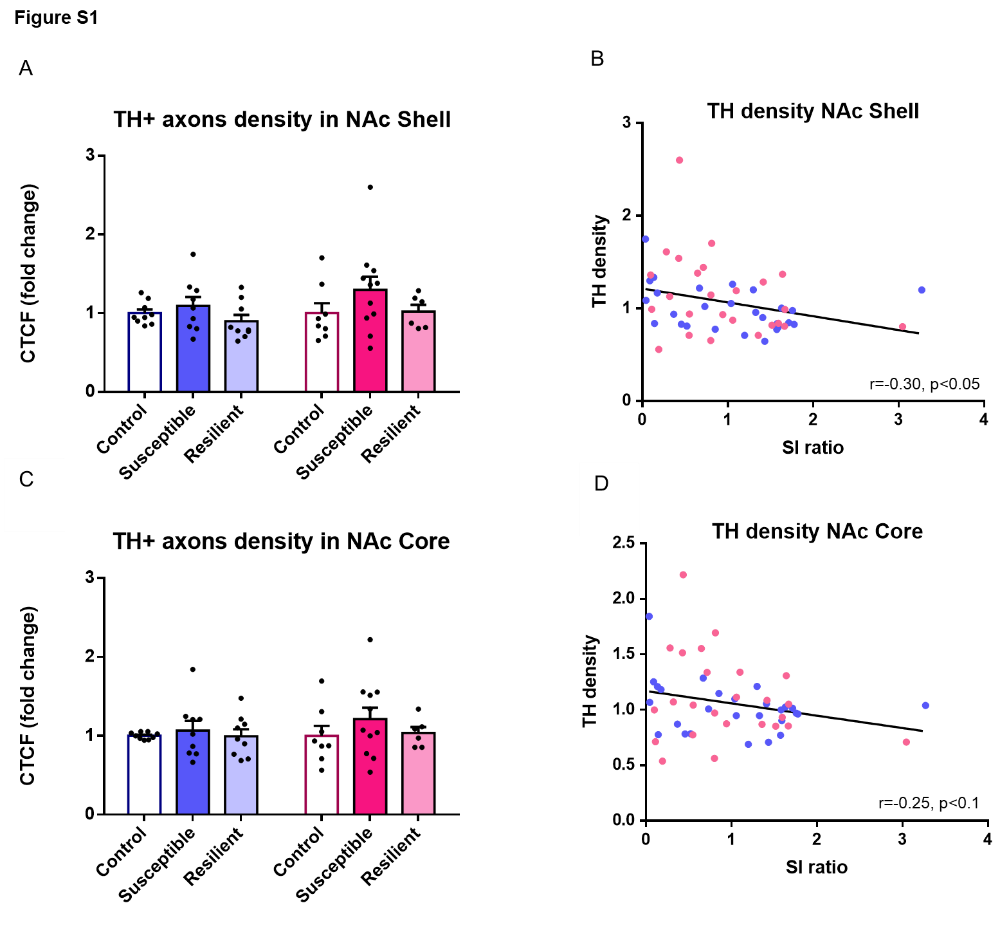
Figure S1.** TH expression in NAc core and shell of social defeated mice (**A**) Fluorescence density of TH+ axons in the NAc shell using ImageJ software, data normalized using corrected total cell fluorescence (CTCF = Integrated Density – (Area of selected cell X Mean fluorescence of background readings)). Female mice showed in pink bar and male mice with blue bar [Two-way ANOVA, Phenotype factor: F_(2,46)_=2.31, *p*=n.s; n=total/male/female, control n=17/9/8, susceptible n=20/9/11, resilient n=15/9/6]. (**B**) Linear correlation between the SI ratio and the CTCF in NAc shell [linear regression, r=-0.30, *p*<0.05, N=52]. (**C**) Fluorescence density of TH+ axons in the NAc shell using ImageJ software, data normalized using CTCF [Two-way ANOVA, Phenotype factor: F_(2,46)_=0.978, *p*=n.s; n=total/male/female, control n=17/9/8, susceptible n=20/9/11, resilient n=15/9/6]. (**D**) Linear correlation between the SI ratio and the CTCF in NAc core [linear regression, r=-0.25, *p*<0.1, N=52]. Bar graphs show mean ± SEM. Data are represented as fold change compared to controls. Each dot represents a single mouse. Values derived from at least three sections per brain. *p≤0.05


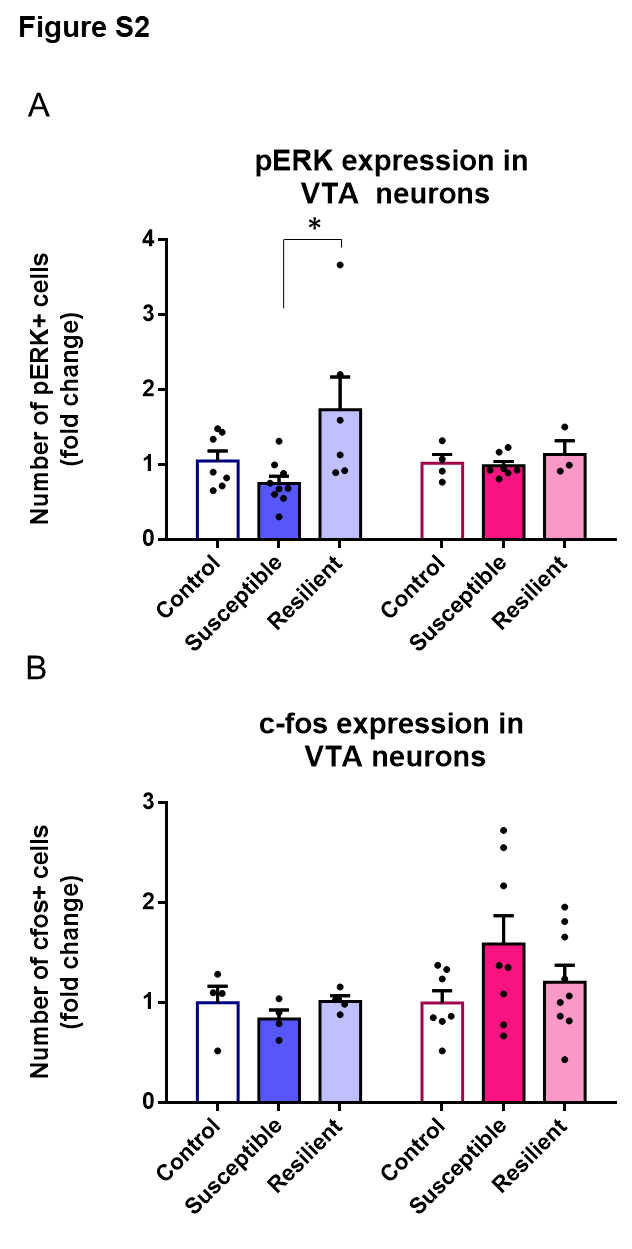
**Figure S2.** pERK and C-fos expression in VTA neurons of social defeated mice (**A**) Stereological count of pERK+ neurons in VTA using ImageJ software [Two-way ANOVA, Phenotype factor: F_(2,30)_=3.35, *p*<0.05; n=total/male/female, Control n=11/7/4, Susceptible n=16/9/7, Resilient n=9/6/3]. (**B**) Stereological count of C-fos+ neurons in VTA using ImageJ software [Two-way ANOVA. Phenotype factor: F_(2,30)_=0.476, *p*=n.s; n=total/male/female, Control n=11/4/7, Susceptible n=12/4/8, Resilient n=13/4/9]. Bar graphs show mean ± SEM. Data are represented as fold change compared to controls. Each dot represents a single mouse. Values derived from at least three sections per brain. *p≤0.05


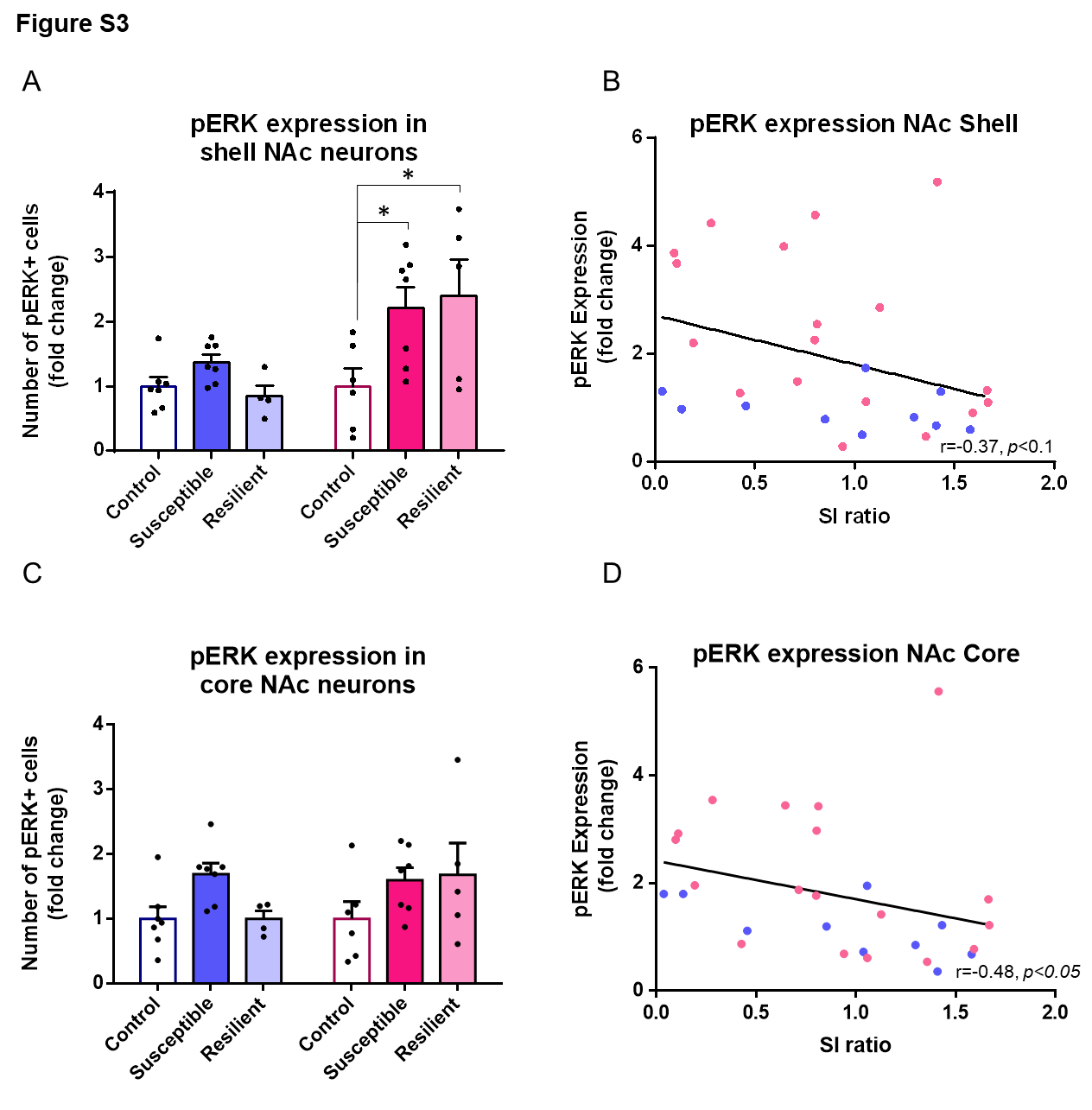
**Figure S3**. pERK expression in NAc core and shell of social defeated mice (**A**) Stereological count of pERK+ neurons in NAc shell using ImageJ software, female mice showed with pink bars and male mice with blue [Two-way ANOVA, Phenotype factor: F_(2,30)_=4.47, *p*<0.05; n=total/male/female, control n=13/7/6, susceptible n=14/7/7, resilient n=9/4/5]. (**B**) Linear correlation between the SI ratio and pERK expression in NAc shell [linear regression, *p*<0.1, r=-0.33, N=28]. (**C**) Stereological count of pERK+ neurons in NAc core using ImageJ software, female mice showed with pink bars and male mice with blue bars [Two-way ANOVA, Phenotype factor: F_(2,46)_=3.67, *p*<0.05; n=total/male/female, control n=13/7/6, susceptible n=14/7/7, resilient n=9/4/5]. (**D**) Linear correlation between the SI ratio and pERK expression in NAc core [linear regression, r=-0.3095, *p*=n.s, N=28]. Bar graphs show mean ± SEM. Data are represented as fold change compared to controls. Each dot represents a single mouse. Values derived from at least three sections per brain. *p≤0.05


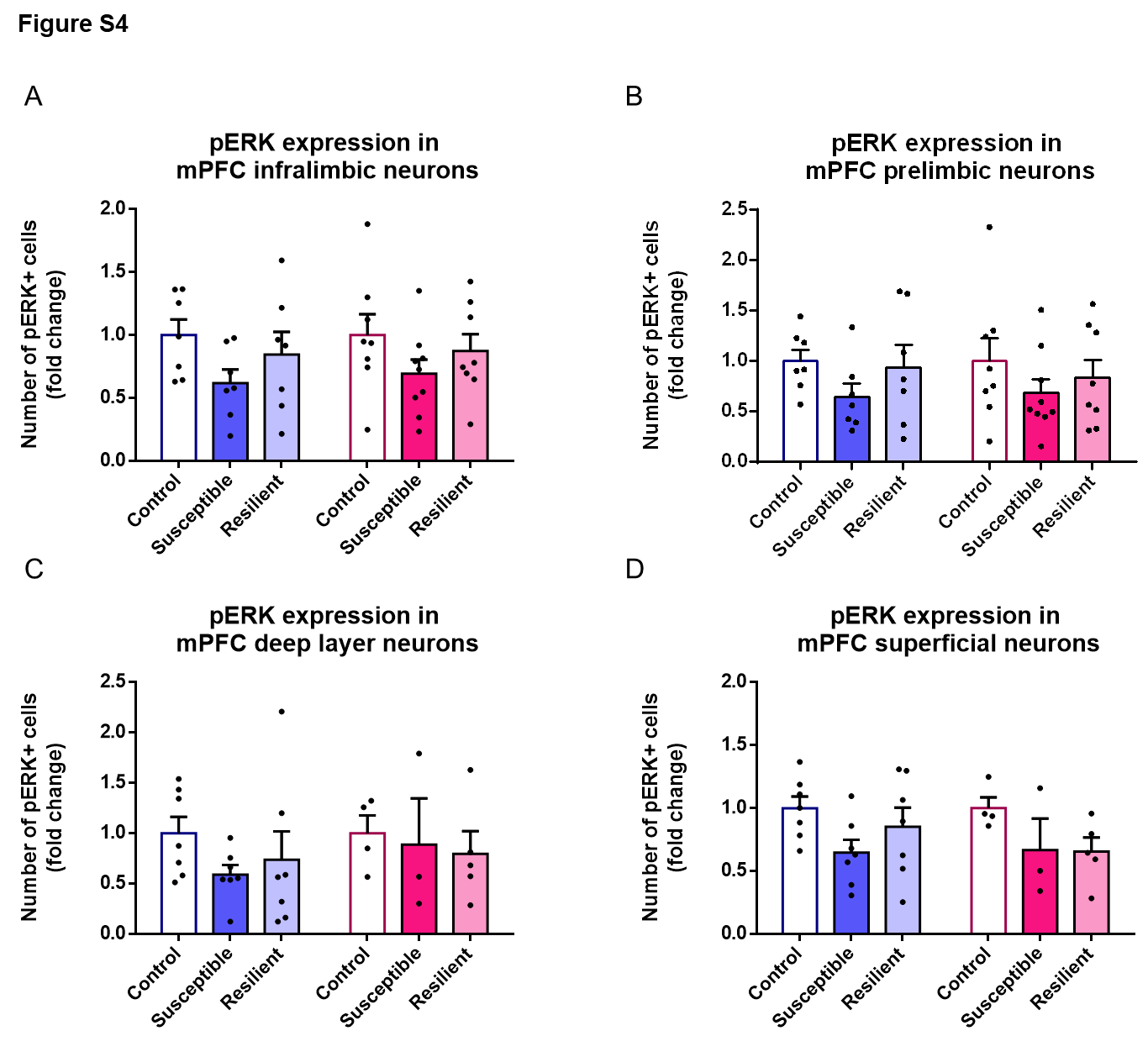
**Figure S4.** pERK expression in different regions of PFC in social defeated mice (**A**) Stereological count of pERK+ neurons in PFC infralimbic using ImageJ software, female mice showed with pink bars and male mice with blue bars [Two-way ANOVA, Phenotype factor: F_(2,40)_=3.10, *p*<0.1; n=total/male/female, control n=15/7/8, susceptible n=16/7/9, resilient n=15/7/8]. (**B**) Stereological count of pERK+ neurons in PFC prelimbic using ImageJ software, female mice showed with pink bars and male mice with blue bars [Two-way ANOVA, Phenotype factor: F_(2,40)_=1.92, *p*=n.s; n=total/male/female, control n=15/7/8, susceptible n=16/7/9, resilient n=15/7/8]. (**C**) Stereological count of pERK+ neurons in deep layer of PFC (layer V and VI) [Two-way ANOVA, Phenotype factor: F_(2,27)_=0.731, *p*=n.s; n=total/male/female, control n=11/7/4, susceptible n=10/7/3, resilient n=12/7/5]. (**D**) Stereological count of pERK+ neurons in superficial layer of PFC (layer II/III and I) [Two-way ANOVA, Phenotype factor: F_(2,27)_=3.26, *p*<0.1; n=total/male/female, control n=11/7/4, susceptible n=10/7/3, resilient n=12/7/5]. Bar graphs show mean ± SEM. Data are represented as fold change compared to controls. Each dot represents a single mouse. Values derived from at least three sections per brain. *p≤0.05


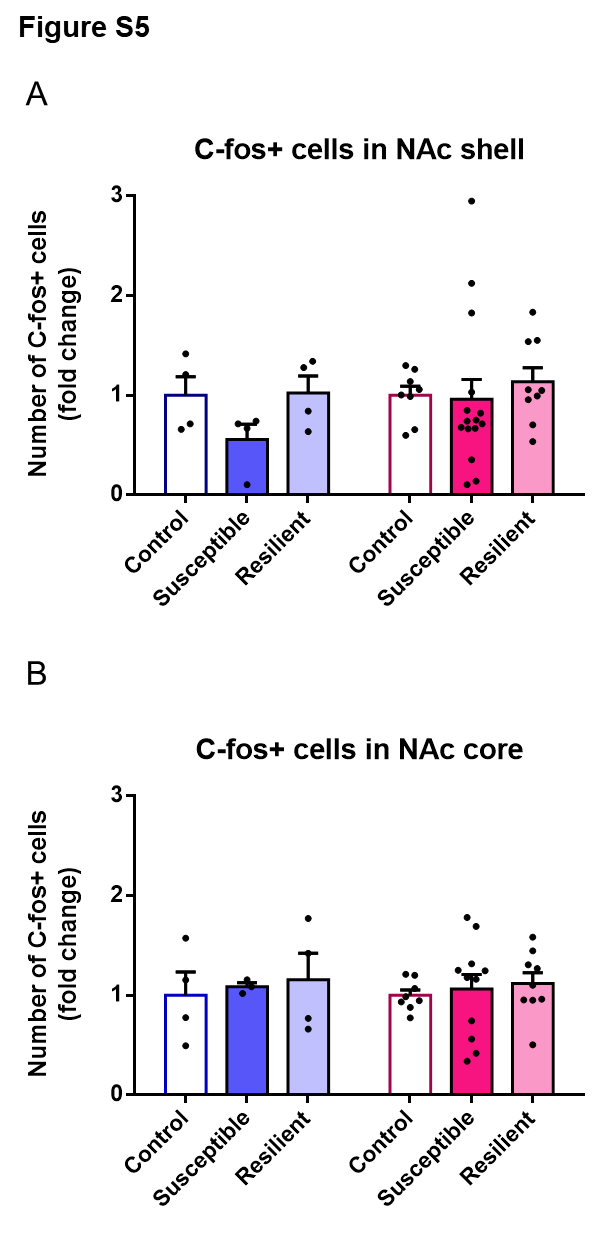
**Figure S5.** C-fos expression in NAc core and shell of social defeated mice (**A**) Stereological count of C-fos+ neurons in NAc shell [Two-way ANOVA, Phenotype factor: F_(2,34)_=0.617, *p*=n.s; n=total/male/female, control n=12/4/8, susceptible n=15/4/11, resilient n=13/4/9]. (**B**) Stereological count of C-fos+ neurons in NAc core [Two-way ANOVA, Phenotype factor: F_(2,34)_=0.349, *p*=n.s; n=total/male/female, control n=12/4/8, susceptible n=15/4/11, resilient n=13/4/9]. Bar graphs show mean ± SEM. Data are represented as fold change compared to controls. Each dot represents a single mouse. Values derived from at least three sections per brain. *p≤0.05


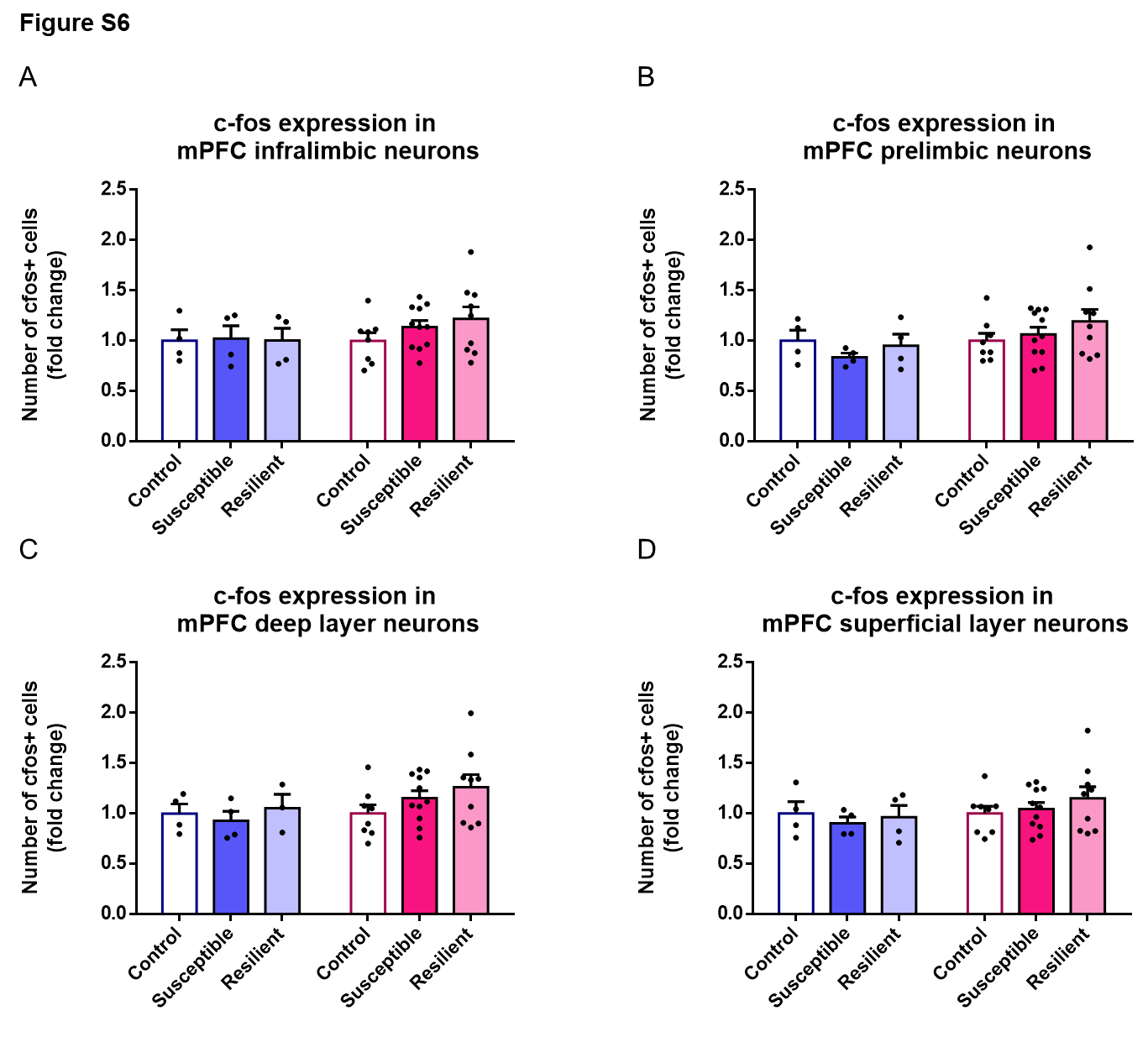
**Figure S6.** C-fos expression in different regions of PFC in social defeated mice (**A**) Stereological count of C-fos+ neurons in PFC infralimbic [Two-way ANOVA, Phenotype factor: F_(2,34)_=0.496, *p*=n.s; n=total/male/female, control n=12/4/8, susceptible n=15/4/11, resilient n=13/4/9]. (**B**) Stereological count of C-fos+ neurons in PFC prelimbic [Two-way ANOVA, Phenotype factor: F_(2,33)_=0.646, *p*=n.s; n=total/male/female, control n=12/4/8, susceptible n=16/4/11, resilient n=12/4/8]. (**C**) Stereological count of C-fos+ neurons in deep layer of PFC (layer V and VI) [Two-way ANOVA, Phenotype factor: F_(2,33)_=0.870, *p*=n.s; n=total/male/female, control n=12/4/8, susceptible n=15/4/11, resilient n=12/4/8]. (**d**) Stereological count of C-fos+ neurons in superficial layer of PFC (layer II/III and I) [Two-way ANOVA, Phenotype factor: F_(2,34)_=0.345, *p*=n.s; n=total/male/female, control n=12/4/8, susceptible n=15/4/11, resilient n=13/4/9]. Bar graphs show mean ± SEM. Data are represented as fold change compared to controls. Each dot represents a single mouse. Values derived from at least three sections per brain. *p≤0.05
